# Tracking of unfamiliar odors is facilitated by signal amplification through anoctamin 2 chloride channels in mouse olfactory receptor neurons

**DOI:** 10.14814/phy2.13373

**Published:** 2017-08-07

**Authors:** Franziska Neureither, Nadine Stowasser, Stephan Frings, Frank Möhrlen

**Affiliations:** ^1^ Department of Animal Molecular Physiology Centre of Organismal Studies, Im Neuenheimer Feld 504 Heidelberg University Heidelberg Germany

**Keywords:** Anoctamin, calcium‐activated chloride channels, odor tracking, olfactory receptor neurons

## Abstract

Many animals follow odor trails to find food, nesting sites, or mates, and they require only faint olfactory cues to do so. The performance of a tracking dog, for instance, poses the question on how the animal is able to distinguish a target odor from the complex chemical background around the trail. Current concepts of odor perception suggest that animals memorize each odor as an olfactory object, a percept that enables fast recognition of the odor and the interpretation of its valence. An open question still is how this learning process operates efficiently at the low odor concentrations that typically prevail when animals inspect an odor trail. To understand olfactory processing under these conditions, we studied the role of an amplification mechanism that boosts signal transduction at low stimulus intensities, a process mediated by calcium‐gated anoctamin 2 chloride channels. Genetically altered *Ano2* ^*−/−*^ mice, which lack these channels, display an impaired cue‐tracking behavior at low odor concentrations when challenged with an unfamiliar, but not with a familiar, odor. Moreover, recordings from the olfactory epithelium revealed that odor coding lacks sensitivity and temporal resolution in anoctamin 2‐deficient mice. Our results demonstrate that the detection of an unfamiliar, weak odor, as well as its memorization as an olfactory object, require signal amplification in olfactory receptor neurons. This process may contribute to the phenomenal tracking abilities of animals that follow odor trails.

## Introduction

When an animal takes up an odor track, an initial chemosensory analysis of the odor source is required to establish the target odor as an olfactory object, and to assign it with subjective hedonic valence (Wilson and Sullivan 2011; Gadziola et al. 2015). A dog trainer, for instance, uses operant‐conditioning methods on a tracking dog, presenting the animal with a plain odor sample, combined with a promise of reward to enforce searching behavior. The dog can memorize the sample as an odor object to aid perception when it encounters traces of the same odor on the track (Wilson and Stevenson 2003; Gottfried 2010; Yeshurun and Sobel 2010; Courtiol and Wilson 2017). This type of sensory learning helps to recognize known objects under difficult conditions. Cognitive processes that promote perceptual stability of an odor, as well as discrimination between different odors, promote identification of a familiar odor object against the otherwise bewildering background that may consist of a large variety of odorants, each at a different concentration. Such processes are performed by the primary cortical area of the olfactory system, the piriform cortex (Barnes et al. 2008; Chapuis and Wilson 2011; Courtiol and Wilson 2017), and they represent the neurobiological underpinning of odor‐guided search. Odor perception is, however, difficult when tracking motivation is low. It appears that, in an environment replete with a great variety of odors, new odors are difficult to smell, while familiar odors are recognized more easily. Thus, the lack of memory severely limits the detection of novel odors, a potentially problematic constraint for animals that explore their environment through the sense of olfaction.

To understand how animals cope with the tracking of unfamiliar odors at weak stimulus intensities, we examined the tracking performance of mice. Focusing on the efficiency of chemoelectrical signal transduction in the olfactory receptor neurons (ORNs) of the nose, we found that a special signal‐amplification mechanism in ORNs is required for tracking novel odors, while tracking familiar odors at the same intensity did not depend on this mechanism. Calcium‐gated chloride channels of the type anoctamin 2 (ANO2, alias TMEM16B) (Schroeder et al. 2008; Stephan et al. 2009; Billig et al. 2011) mediate this amplification process, as demonstrated by comparing wild‐type with ANO2‐deficient (*Ano2* ^*−/−*^) mice. We found that ANO2 amplified the afferent olfactory signal specifically at low odor concentrations. Amplification enabled cognitive processing of weak odor stimuli and promoted the learning process that established an unfamiliar odor as a memorized odor object. Our results demonstrate that the problem of missing memory content in the tracking of weak, unfamiliar odors can be solved by peripheral signal amplification, a process that potentiates olfactory performance under natural conditions.

## Methods

### Ethical approval

Male, 10‐ to 14‐week‐old C57BL/6N mice (Charles River Laboratories, Germany) and *Ano2* ^*−/−*^ mice (Billig et al. 2011) (MGI: 5052299; Ano2^tm1.1Tjj^; kindly provided by Thomas Jentsch, Leipniz‐Institute for Molecular Pharmacology, Berlin) were kept in groups of 2–3 with ad libitum access to food and water. A standard 12‐h light/dark cycle was provided (light on: 7 am to 7 pm), temperature was maintained at ±22°C at a relative humidity of 40–50%. Female mice were not used in this study to avoid odor‐sensitivity fluctuations during the estrous cycle (Kumar and Archunan 1999). All experiments were approved by the Regierungspräsidium Karlsruhe and were in agreement with national and international guidelines. All steps were taken to minimize the animals’ pain and suffering. For general health screening, a modified version of the SHIRPA test (Rogers et al. 1997) was used for both genotypes. Spontaneous activity, anxiety, body strength, and muscle tone as well as several reflexes were tested according to the provided scoring system. Both wild‐type and *Ano2* ^*−/−*^ mice showed normal neurophysiological parameters. General behavior was monitored using the *LABORAS* animal behavior observation system (Metris B.V., the Netherlands) individually for six mice of each genotype for 72 h. The test results on specific activities (locomotion, climbing, rearing, grooming, eating, drinking) did not show any significant differences between wild‐type and *Ano2* ^*−/−*^ mice. Following the conclusion of this study, animals were killed by an overdose of isoflurane.

### Behavioral assays

To increase tracking motivation, mice were food deprived 3 days prior to testing. The body weight was adjusted to 90% of the individual normal weight and stabilized by limiting food to 2–3 g/day for each mouse. Food was given after the daily tracking test. At the end of the testing phase, animals were returned to ad libitum feeding. Free access to water was always ensured. Each mouse was tested for six consecutive days with one test conducted each day. On the last day, the mice performed a motivational control test, with the reward not hidden but exposed to view (visual tracking control). This control was a necessary test for search motivation in animals that were unable to track olfactory cues. On the day before the first test, mice were given sucrose pellets (Bio Neutral Globuli, Flora Cura, London, UK) to introduce the reward and to establish its appetitive effect on each individual animal. Video‐taped tracking tests (Sygnis Tracker, Sygnis, Heidelberg, Germany) were conducted in a test arena, a polycarbonate Macrolon 3H box (42.5 × 26.6 × 18 cm) filled with approximately 3 cm of bedding material (LT‐E‐001, ABEDD, Wien, Austria) that was changed for each mouse. The arena box was placed between opaque plastic walls to impede visual orientation. Before testing, each mouse was placed into the arena for a 10‐min habituation phase without any odor cue, to allow the animal to habituate to the test arena. The mouse was then removed and the arena was prepared for the tracking test. A plastic box (paraffin embedding box from Histosette 1, M 491‐2, Simport, Beloeil, Quebec, Canada) with two compartments (30 × 25 mm and 10 × 25 mm) was used. The larger compartment could be closed with a perforated lid and housed the olfactory cue: six sucrose pellets each soaked with 5 *μ*L odor solution. The lid was closed so that the odorized pellets were not accessible to the mouse, while the odor could diffuse out of the box and reach the surface of the bedding. The smaller compartment had no lid and was filled with six nonodorized sucrose pellets as food reward. The box was buried 1 cm under the surface of the bedding at randomized places of the arena in every trial. Boxes with nonodorized pellets were not found by the mice, as established by test runs before setting up the experiments. Unsuccessful trials were aborted after 300 sec, and animals were returned to their home cage without reward. Consequently, a tracking time of 300 sec indicates that the mouse did not find the reward. To identify a threshold odor intensity in preparatory trials for this experiment, we lowered the syringol concentration in the odor solution until ~50% of syringol‐naïve wild‐type mice successfully tracked the odor cue upon their first contact with syringol. This solution was 0.03 mg/mL syringol in water placed onto the six pellets (6 × 5 *μ*L) within the closed compartment. For tracking experiments, mice were placed onto a starting position on one side of the arena. The tracking time was then measured until the animal discovered and excavated the box. To enforce tracking behavior in subsequent trials, mice were allowed to eat the reward. After returning each mouse to its home cage, the arena was cleaned with 70% ethanol and then charged with fresh bedding material for the next trial. Each animal performed one trial per day on five consecutive days. Tracking times obtained from nine male, age‐matched mice were averaged and are presented with SEM in the graphs.

### Electroolfactography

For ex vivo electrical recording from the olfactory epithelium, mice were anesthetized by inhalation of isoflurane (0.5 mL in 2 L air) (Baxter Healthcare, Deerfield, IL) and killed by an isoflurane overdose (0.5 mL in 0.25 L air). To expose the olfactory epithelium, the fur was removed from the skull, and the head was then opened along the sagittal suture. The nasal septum was carefully removed to expose the olfactory turbinates with the chemosensory epithelium. EOG recordings were always taken from the same area of the right nasal cavity: the dorsomedial surface of turbinate IIa (Ressler et al. 1993) (location II.4 in Coppola et al. 2013), a zone that projects to the dorsolateral side of the olfactory bulb. The preparation was placed into an interface chamber, which was mounted on a vibration‐isolating table and shielded from electrical fields by a Faraday cage. Hydration of the epithelium was ensured by directing a constant (0.15 L/min) stream of humidified and deodorized air through an odor tube onto the tissue surface (distance of tube opening: 1.5 cm). For stimulation, 20 msec odor pulses were injected into the air stream from a test tube using a computer‐controlled pneumatic pico pump (PV830, WPI, Sarasota, FL) at a pressure of 8 psi. Electrical responses of the olfactory epithelium were recorded through a micropipette with an Ag/AgCl electrode immersed into pipette solution (145 mmol/L NaCl, 5 mmol/L KCl, 1 mmol/L MgCl_2_, 1 mmol/L CaCl_2_; 10 mmol/L HEPES, pH 7.4). Micropipettes were pulled to a resistance of 1.5–2.5 MΩ from borosilicate glass capillaries (GP150F‐10, 0.86 × 1.50 × 100 mm, Science Products GmbH, Hofheim, Germany) using a Flaming–Brown puller (Sutter Instruments, Novato, CA). The open‐pipette resistance was determined for each micropipette. A grounding electrode (AgCl pellet, World Precision Instruments, Sarasota, FL) was placed onto the preparation, and electrical contact was provided by Ringer's solution (140 mmol/L NaCl, 5 mmol/L KCl, 1 mmol/L MgCl_2_, 1 mmol/L CaCl_2_, 10 mmol/L d‐glucose, 1 mmol/L Na‐pyruvate, 10 mmol/L HEPES, pH 7.4). EOGs were recorded as local surface potentials using a differential amplifier (DP‐301, Warner Instruments, Hamden, CT) at a gain of 100×. Signals were digitized (BNC‐2120, National Instruments, Munich, Germany) and processed using WinWCP (version 4.6.1, provided by the University of Strathclyde, Glasgow, UK). EOGs were sampled at a rate of 10 kHz and low‐pass filtered at 1 kHz. Offline analysis was conducted using the software Origin (version 9.0, OriginLab Corp., Northampton, MA). Room temperature was 20–25°C and the recordings were always conducted during the same time of the day in order to rule out any circadian influences.

### Olfactory stimulation

Syringol (1,3‐dimethoxy‐2‐hydroxybenzene; 2,6‐dimethoxyphenol; D135550, Sigma‐Aldrich, Munich, Germany) was chosen as a food‐related (Burdock 2010), attractive odorant, which is soluble in water to some extent. Two milliliter of syringol solution (0.5 mg/mL, 1 mg/mL, 3 mg/mL, or 7 mg/mL) was filled into the test tube (ecoLab round‐bottom centrifuge tube, 100 × 16 mm NeoLab, Heidelberg, Germany). A partition equilibrium between the aqueous phase and the headspace air, which had a volume of 11.5 mL, was usually achieved within 1 min, but a minimum of 5 min was routinely observed before starting an experiment. A 20 msec pulse at 8 psi injected reproducibly 4 mL of the headspace air into the odor tube. The local odor concentration at the recording spot was not determined. Maximal intensity responses were recorded with either of two stimuli: The odor‐mix Henkel 100, which consists of 100 different odorants (Henkel 100, kindly provided by Dr. Thomas Gerke, Henkel KGaA, Düsseldorf, Germany), was used at 1:1000 dilution (2 mL in the test tube) to stimulate all ORNs. Alternatively, in experiments with different concentrations of syringol, 9 mg of solid syringol was placed in the test tube to maximally stimulate all ORNs capable of responding to syringol.

In experiments where the Cl^−^ channel blocker niflumic acid (300 *μ*mol/L in pipette solution; N0630, Sigma‐Aldrich) (Nickell et al. 2006; Sagheddu et al. 2010) or the Ca^2+^ chelator ethylene glycol‐*bis*(‐aminoethylether)‐*N*,*N*,*N′*,*N′*‐tetraacetic acid (10 mmol/L in Ca^2+^‐free pipette solution; EGTA, E3889, Sigma‐Aldrich) were used (Fig. 2), EOGs were recorded as follows. (1) *Test for EOG block by niflumic acid*: First, a 5‐*μ*L drop of pipette solution without niflumic acid was positioned at the recording site, and the control EOG was recorded with a 20‐ms pulse of odor mix from the odor tube. Second, a 5‐*μ*L drop of pipette solution containing 300 *μ*mol/L niflumic acid was placed on the recording spot, and the test EOG was induced using the same odor stimulus. EOG recordings were normalized to the control trace and averaged over five animals. (2) *Test for the effect of EGTA*: First, a 5‐*μ*L drop of pipette solution containing 1 mmol/L Ca^2+^ was positioned at the recording site, and a standard EOG was recorded with a 20‐msec pulse of odor mix from the odor tube. Second, a pipette filled with Ca^2+^‐free pipette solution containing 10 mmol/L EGTA was positioned for recording, and the control EOG was recorded with the same odor stimulus. Third, 5 *μ*L drop of Ca^2+^‐free pipette solution was placed on the recording spot, and the Ca^2+^‐free test EOG was recorded. EOG amplitudes were normalized to the control recording and averaged over nine animals.

In all EOG experiments with syringol (Figs. 3 and 4), no solution was deposited on the epithelial surface. For single‐response experiments (Fig. 3), seven 20 msec syringol pulses were applied at the indicated concentration with interstimulus intervals of 240 sec, and the recorded EOGs were averaged. To determine the response intensity of these average traces, we calculated the trace integral over 2 sec, a parameter that yielded a more reliable EOG quantification than the maximal amplitudes. For the triple‐pulse experiments (Fig. 4), the interstimulus intervals were shortened to ≤5 sec. Each test consisted of three 20 msec pulses applied at one interstimulus interval. Two tests were recorded for each interstimulus interval and averaged. After each triple pulse, voltage traces were allowed to return to baseline. All indicated interstimulus intervals (from 0.25 to 5 sec) were applied in a series of tests on the same animal. A recovery time of 60 sec was allowed following each test because full response amplitudes of the second pulse were obtained after this time period. Results from 3 to 9 animals were then averaged and presented with SEM.

### Statistical analysis

Statistics were calculated and the graphs were prepared using OriginLab 9.0 (OriginLab Corporation, Northampton). The Gaussian distribution of data was determined by first applying the Shapiro–Wilk test. If the data were normally distributed, a standard two‐tailed unpaired Student's *t* test was calculated. A Mann–Whitney's *U* test was used for data that did not meet the normal distribution criteria. Besides, a heteroscedastic two‐tailed unpaired Student's *t* test was used on the test dataset, if the control dataset was normalized (Fig. 2E). To test for influences of different factors on and interaction between data, a two‐way ANOVA was applied followed by a Bonferroni correction (post hoc test). The *F* values are given in the figure legends to describe the data variance. As a predetermined significance threshold for all tests described above, a *P* < 0.05 was determined (*P < 0.05, **P < 0.01, ***P < 0.001). All data, if not indicated otherwise, are displayed as mean ± SEM.

## Results

### Odor tracking in naïve and experienced mice

**Figure 1:**
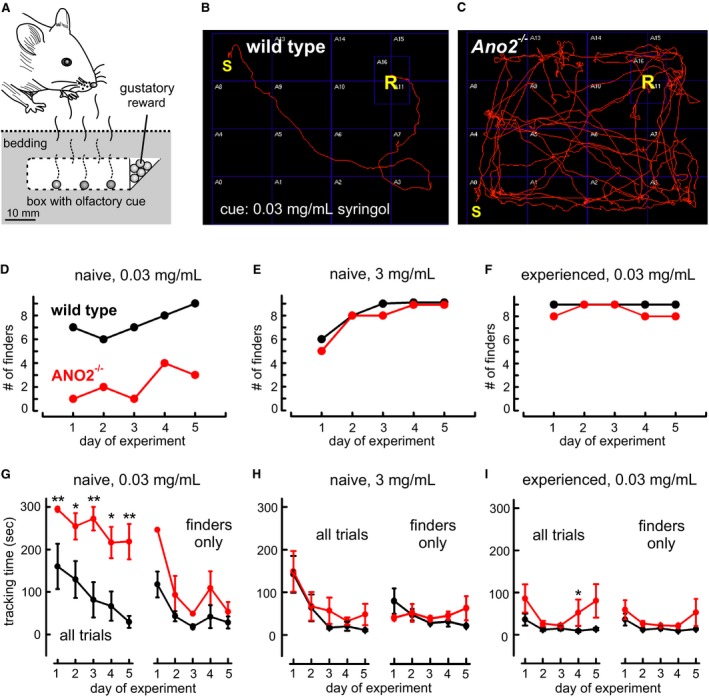
Impaired tracking performance in *Ano2* ^*−/−*^ mice. (A) Sketch of the behavioral tracking experiments. Mice were guided by an olfactory cue to a gustatory reward (odorless sucrose pellets), hidden under bedding material. A locked, perforated compartment contained six odorized sucrose pellets to produce the olfactory cue. (B) Video trace of a wild‐type mouse during the search for the reward after 5 days of testing. The search time from start (S) to discovery of the reward (R) was 15 sec. The olfactory cue originated from 30 *μ*L syringol solution (0.03 mg/mL). Indicated squares are 12 cm × 12 cm, the thickness of the bedding layer was approximately 3 cm. (C) Video trace of an *Ano2* ^*−/−*^ mouse on the 5th day of testing under the same conditions. The animal explored the arena for 300 sec without discovering the reward. (D) Numbers of successful finders in cohorts of nine syringol‐naïve wild‐type and nine syringol‐naïve *Ano2* ^*−/−*^ mice at near‐threshold stimulus intensity (0.03 mg/mL) tested over five consecutive days. (E) Numbers of successful finders out of nine initially syringol‐naïve animals in tracking a robust odor cue (3 mg/mL syringol). (F) Numbers of successful finders at threshold stimulus intensity with nine mice of each genotype experienced in syringol tracking. (G) Tracking times of mice from (D). Mean search time required on each day to find the reward during experiments at near‐threshold intensity with syringol‐naïve mice. The maximal search time allowed (300 sec) was assigned to mice that did not find the reward. For the successful finders, search times are depicted on the right. (H) Tracking times of mice from (E). Mean search times of syringol‐naïve mice at robust stimulation (left: all mice, right: finders only). (I) Tracking times of mice from (F). Fast tracking by syringol‐experienced mice of both genotypes at threshold concentration (left: all mice, right: finders only). Mean ± SEM, Mann–Whitney *U* test; *P < 0.05, **P < 0.01.

To examine tracking performance, mice were allowed to search for sucrose pellets hidden under a layer of bedding. To guide the animals toward the reward, an olfactory cue was positioned adjacent to the sucrose pellets in a perforated box (Fig. 1A). The odorant syringol, which smelled like unsmoked bacon to humans, was used as food‐related odor after we found in preparative experiments that this compound acted as an appetitive stimulus. Wild‐type mice eagerly sniffed out syringol samples, and the threshold concentration for syringol tracking (50% tracking success within 300 sec) was 0.03 mg/mL for originally syringol‐naïve mice (Fig. 1B and Supplemental Movie S1). In contrast, syringol‐naïve ANO2‐deficient *Ano2* ^*−/−*^ mice (Billig et al. 2011) did not find the sucrose pellets at that concentration (Fig. 1C and Supplemental Movie S2). Thus, ANO2‐type Ca^2+^‐gated chloride channels, which amplify odor transduction in olfactory receptor neurons (ORNs) (Kleene 1993; Kurahashi and Yau 1993; Lowe and Gold 1993; Stephan et al. 2009; Billig et al. 2011), appear to be essential for tracking performance. In repeated experiments over the course of 5 days, originally syringol‐naïve mice were monitored while learning to follow a syringol trail to the sucrose pellets. The number of successful finders among nine animals of each genotype increased over the course of 5 days from 6–7 to 9 in wild‐type mice, but only from 1 to 3 in *Ano2* ^*−/−*^ mice (Fig. 1D). In contrast, both syringol‐naïve genotypes performed similarly well with a robust odor stimulus (3 mg/mL syringol), and all animals eventually learned to find the reward (Fig. 1E). Moreover, mice that had already learned to follow a 3 mg/mL syringol cue reliably in our 5‐day training protocol also found the reward even at the low concentration of 0.03 mg/mL syringol, and *Ano2* ^*−/−*^ mice performed almost as well as wild‐type mice (Fig. 1F). These results demonstrate that *Ano2* ^*−/−*^ mice displayed impaired tracking performance when the odor cue is *both* weak *and* unfamiliar. With *either* robust *or* familiar odor cues, both genotypes performed similarly well.

The times required for tracking the odor cue depended on odor intensity and tracking experience. Figure 1G illustrates that the originally syringol‐naïve wild‐type animals (same mice as in Fig. 1D) decreased their tracking time from 160 ± 53 sec to 29 ± 14 sec (*n* = 9) within 5 days. When only the successful finders in this group were considered for each day, the mean tracking time decreased from 130 ± 31 sec to 29 ± 14 sec (Fig. 1G, *black lines*). The nine syringol‐naïve *Ano2* ^*−/−*^ mice needed on average more time (218 ± 41 sec on the 5th day). Only one third of this cohort was eventually able to find the reward, requiring on average 255 sec (1st day, 1 animal) to 55 ± 22 sec (5th day, three animals) for the task (Fig. 1G, *red lines*). Thus, a significant phenotype of the *Ano2* ^*−/−*^ mouse line revealed itself in the tracking success and the tracking speed at threshold odor concentration. In contrast to their performance at odor threshold, syringol‐naïve animals of both genotypes rapidly learned to track robust syringol cues (Fig. 1H, 3 mg/mL, same mice as in Fig. 1E). Mean tracking times of wild types decreased from 142 ± 43 sec to 11 ± 2.5 sec (*n* = 9) over the course of 5 days (finders only: 63 ± 26 sec to 11 ± 2.5 sec), while *Ano2* ^*−/−*^ mice performed almost as well (all nine: 149 ± 48 sec to 48 ± 25 sec; finders only: 28 ± 5 sec to 48 ± 25 sec; Fig. 1H, *red lines*). Syringol‐experienced mice of both genotypes, which had previously learned to track 3 mg/mL syringol, had no problems with finding the reward rapidly even at 0.03 mg/mL syringol (Fig. 1I, same mice as in Fig. 1F). Wild types decreased their search time over 5 days from 36 ± 14 sec to 13 ± 3 sec (*n* = 9; all finders) (Fig. 1I, *black lines*), while *Ano2* ^*−/−*^ mice required slightly more time (86 ± 33 sec to 80 ± 40 sec, *n* = 9; finders only: 59 ± 22 sec to 53 ± 32 sec) (Fig 1I, *red lines*). Thus, both wild‐type and *Ano2* ^*−/−*^ mice improved their tracking ability through experience and both genotypes were able to track 0.03 mg/mL syringol. However, memorizing the originally unfamiliar odor required a stronger stimulus than tracking the familiar odor and, for most *Ano2* ^*−/−*^ mice, the near‐threshold syringol concentration was too weak to acquire specific odor memory on syringol. These results suggest that ANO2‐mediated signal amplification promotes odor learning specifically at low stimulus intensities.

### Afferent signal amplification by anoctamin 2

**Figure 2:**
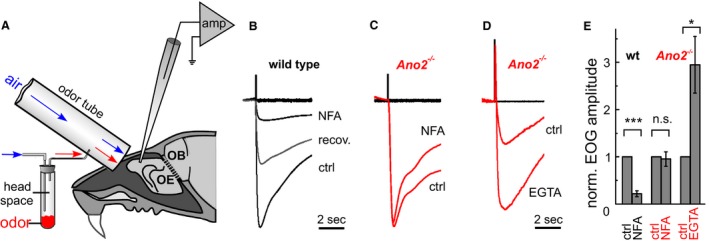
Electroolfactogram recording from mouse olfactory epithelium. (A) Recording configuration for turbinate II of the olfactory epithelium (OE). Odor pulses were injected from the headspace of a test tube into the airstream of the odor tube. OB: olfactory bulb; amp: differential amplifier (B) Transient negative surface potentials (normalized EOGs from wild type) elicited by a 20‐msec pulse of odor mix (Henkel 100, 1:1000) with 5 *μ*L pipette solution deposited at the recording site. Niflumic acid (NFA, 300 *μ*mol/L) inhibited the EOG when added to the pipette solution. After a few minutes, partial recovery was observed (middle trace) as niflumic acid diffused away from the recording spot through the mucus layer. (C) A normalized EOG triggered by odor mix recorded from turbinate II of an *Ano2* ^*−/−*^ mouse without (*ctrl*) and with 300 *μ*mol/L NFA. (D) Normalized EOGs from an *Ano2* ^*−/−*^ mouse with 5 *μ*L pipette solution (*ctrl*) and with Ca^2+^‐free Ringer′s containing 10 mmol/L EGTA deposited on the recording area. (E) EOG amplitudes, normalized to each control recording, show NFA sensitivity in wild‐type mice (fractional reduction of EOG to 0.22 ± 0.06; *n* = 5), NFA insensitivity in *Ano2* ^*−/−*^ mice (fractional reduction of EOG to 0.95 ± 0.15; *n* = 5), and the release from Ca^2+^ inhibition in *Ano2* ^*−/−*^ mice (EOG increase by a factor of 2.95 ± 0.6; *n* = 9). All results are mean ± SEM; heteroscedastic two‐tailed unpaired Student's *t*‐test, *P < 0.05, ***P < 0.001.

To understand the reduced tracking performance of *Ano2* ^*−/−*^ mice, we analyzed the afferent signal generation in the olfactory epithelium. Odor‐induced electroolfactograms (EOGs) (Scott and Scott‐Johnson 2002; Nickell et al. 2007) of wild‐type mice were recorded from the dorsomedial surface of turbinate II.4 (Coppola et al. 2013) in fresh preparations of the nasal cavity (Fig. 2A). The recording site was well exposed to the stimulus‐carrying air flow, was not prone to surface liquid accumulation, and produced fairly consistent EOGs in different animals.

EOGs were transient negative potentials, largely suppressed by 300 *μ*mol/L niflumic acid, a broad spectrum Cl^−^ channel blocker (normalized EOGs in Fig. 2B,E). In contrast, EOGs recorded from *Ano2* ^*−/−*^ mice were not significantly affected by niflumic acid (normalized EOGs in Fig. 2C,E), indicating a transduction process without Cl^−^ channel contribution. This finding is consistent with a previous report on the absence of any Ca^2+^‐gated Cl^−^ currents from isolated ORNs of *Ano2* ^*−/−*^ mice (Billig et al. 2011). Stimulation of ORNs also opens cAMP‐gated Na^+^/Ca^2+^‐permeable (CNG) channels, and one of their indicative properties is an apparent block by extracellular Ca^2+^ ions (Kleene 1995; Dzeja et al. 1999). When we reduced the surface Ca^2+^ concentration near the pipette tip by using a Ca^2+^‐free pipette solution with the Ca^2+^ chelator EGTA, the signal increased significantly, presumably reflecting the release of CNG channels from Ca^2+^ blockage (Fig. 2D, E). These results suggest that EOGs in *Ano2* ^*−/−*^ mice originate from CNG channels and not from a CNG/Cl^−^ channel combination as in wild‐type mice (Reisert et al. 2005; Li et al. 2016).

**Figure 3:**
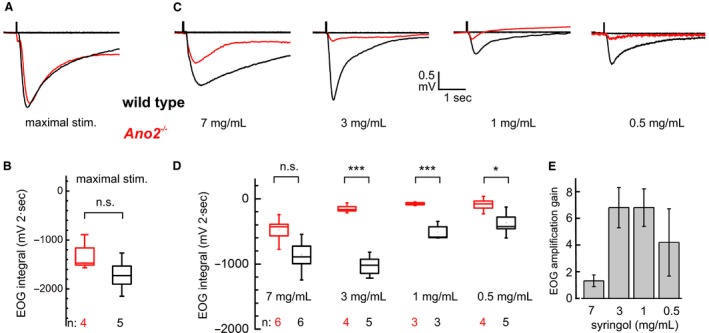
Concentration dependence of signal amplification. (A) Representative EOG traces recorded with robust syringol stimuli from a wild‐type (*black trace*) and an *Ano2* ^*−/−*^ mouse (*red trace*). For stimulation, a 20‐msec pulse of air was injected from the headspace of a test tube containing 9 mg undissolved syringol. (B) EOG response intensities, measured as integrals of syringol‐induced transients over 2 sec, do not differ significantly between wild‐type (1718 ± 185 mV · 2 sec; *n* = 4; *black*) and *Ano2* ^*−/−*^ mice (1336 ± 175 mV · 2 sec; *n* = 5; *red*); Mann–Whitney *U* test; *P* = 0.15. (C) EOG recordings from wild‐type mice (*black*) and *Ano2* ^*−/−*^ mice (*red*) are different at syringol concentrations that elicit submaximal responses. (D) At submaximal syringol concentrations, mean EOG integrals from wild‐type mice (*black*) are larger than EOGs from *Ano2* ^*−/−*^ mice (*red*); two‐tailed unpaired student's *t*‐test; *P < 0.05, ***P < 0.001. Bottom numbers indicate number of animals tested. The concentration dependence over the tested range varied significantly between genotypes, as brought out by two‐way ANOVA followed by Bonferroni post hoc correction: all means, *F*_1,34_ = 16.7, *P* < 0.001; concentration dependence, *F*_3,34_ = 13.1, *P* < 0.0001; interaction, *F*_3,34_ = 3.8, *P* < 0.05. (E) Amplification gain of the ANO2‐mediated mechanism expressed as the ratio of wild‐type EOG integrals to *Ano2* ^*−/−*^ EOG integrals from (D) for each syringol concentration. This analysis brings out a > 6‐fold amplification at submaximal odor concentrations. Ratios are shown with relative errors.

As behavioral deficits in *Ano2* ^*−/−*^ mice occurred only at low stimulus intensities, we compared EOGs in response to weak and strong syringol stimulations. The strongest odor intensity was achieved by injecting a 20 msec pulse of air from the headspace above 9 mg of solid syringol into the air stream of the odor tube. This stimulus elicited EOG responses of similar amplitudes and time courses in both genotypes (Fig. 3A). The averaged EOG time integrals, *∫V*_*EOG*_(*t*)d*t* over the initial 2 sec of the EOG, did not differ significantly (Fig. 3B). This surprising result demonstrates that the maximal afferent signal in the absence of ANO2 has the same amplitude as the maximal signal with ANO2 present. A similar observation was reported earlier for air‐phase EOGs obtained with strong stimuli containing 8–17 different odorants (Billig et al. 2011). These findings demonstrate that responses to strong olfactory stimulations are not further amplified by the ANO2 mechanism. In contrast, EOGs recorded at submaximal syringol intensities were indeed amplified. When delivered at 7, 3, 1, or 0.5 mg/mL, syringol stimuli generated responses smaller than maximal (Fig. 3C), and significant differences between wild‐type and *Ano2* ^*−/−*^ mice emerged at 3, 1, and 0.5 mg/mL syringol (Fig. 3D). The EOG ratios (wild type/*Ano2* ^*−/−*^) demonstrate that a sixfold amplification of submaximal response intensities is associated with the expression of ANO2 (Fig. 3E).

### Temporal acuity and anoctamin 2

**Figure 4:**
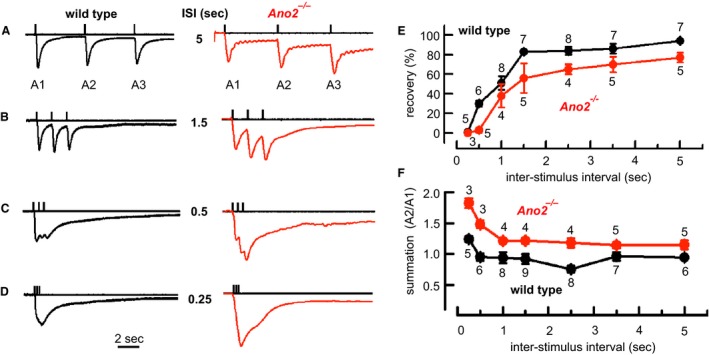
Contribution of ANO2 to dynamic properties of the EOG response. (A) EOG responses to three 20 msec pulses of syringol (3 mg/mL in the test tube) delivered at an interstimulus interval (ISI) of 5 sec recorded from the olfactory epithelium of a wild‐type (*black*) and an *Ano2* ^*−/−*^ mouse (*red*). Each trace was normalized to its first peak amplitude. (B–D) Similar recordings with interstimulus intervals of 1.5, 0.5, and 0.25 sec. (E) Signal recovery, measured as the percent decline of the first EOG potential before the onset of the second, differs systematically between wild‐type and *Ano2* ^*−/−*^ mice over the entire range of interstimulus intervals. Two‐way ANOVA followed by Bonferroni post hoc correction: all means, *F*_1,80_ = 24.2, *P* < 0.0001; dependence on ISI duration, *F*_6,80_ = 48.6, *P* < 0.0001; interaction, *F*_6,80_ = 0.83, *P* = 0.55; *n* = 3–9 (mean = 5.7). (F) As a measure of response summation, the increase of the second response amplitude with respect to the first, is more pronounced in *Ano2* ^*−/−*^ mice than in wild types over the entire range of interstimulus intervals. Two‐way ANOVA followed by Bonferroni post hoc correction: all means, *F*_1,78_ = 73, *P* < 0.0001; dependence on interstimulus intervals, *F*_6,78_ = 9.3, *P* < 0.0001; interaction, *F*_6,78_ = 1.9, *P* = 0.09; *n* = 3–9 (mean = 5.6). Numbers of test animals are indicated at each data point.

In mice, tracking behavior involves repetitive sniffing, an accelerated breathing pattern believed to optimize odor uptake into the nose and to entrain smell‐related cerebral processes to the respiratory cycle (Shusterman et al. 2011; Fukunaga et al. 2012; Tsanov et al. 2014). To find out whether *Ano2* ^*−/−*^ mice show an altered EOG response to repetitive stimulation, we applied three 20 msec odor pulses at various interstimulus intervals. Within each triple‐pulse protocol, EOGs from *Ano2* ^*−/−*^ mice recovered less completely than wild‐type EOGs when stimulated at interstimulus intervals from 0.25 to 5 sec (Fig. 4A–D). EOG recovery was significantly faster in the wild‐type compared to *Ano2* ^*−/−*^ mice (Fig. 4E). The residual signal in *Ano2* ^*−/−*^ mice promoted summation, leaving the second response consistently larger than the first (Fig. 4F). These results indicate a reduced capacity of the olfactory epithelium in *Ano2* ^*−/−*^ mice to produce separated responses to individual stimuli during repetitive stimulation at typical sniffing frequencies. As nonseparated responses are indicative of reduced temporal resolution in the afferent signal, it appears that the afferent signals tend to be blurred in the absence of ANO2.

## Discussion

Our results suggest that the ANO2‐mediated amplification mechanism enables mice to track weak, unfamiliar olfactory cues. Amplification takes place in the chemosensory cilia of ORNs where odorant receptors bind odorants (Buck and Axel 1991) and activate adenylate cyclase to synthesize cAMP as a diffusible messenger. This triggers opening of CNG channels (Nakamura and Gold 1987) and ANO2‐type Ca^2+^‐gated Cl^−^ channels (Kleene and Gesteland 1991; Kurahashi and Yau 1993; Lowe and Gold 1993; Stephan et al. 2009). CNG channels provide initial depolarization and Ca^2+^ influx, whereas Cl^−^ channels boost depolarization through Cl^−^ efflux (Reisert et al. 2003, 2005; Dibattista et al. 2017). ANO2 channels increase the amplitude of transduction currents at submaximal odor stimuli and, in addition, accelerate recovery as they rapidly close when intracellular Ca^2+^ returns to submicromolar levels (Reisert and Matthews 1998; Reisert et al. 2003; Stephan et al. 2012). Both factors are crucial for the encoding precision of the olfactory epithelium (Bhandawat et al. 2005, 2010; Ghatpande and Reisert 2011). We interpret the results from our experiments with *Ano2* ^*−/−*^ mice in terms of an ORN dysfunction that causes an afferent signal compromised with respect to signal intensity and temporal resolution.

Although expression of ANO2 is sparse, contributions to the odor‐tracking phenotype from other brain areas cannot be excluded for the *Ano2* ^*−/−*^ line. Specifically, ANO2 plays a role in motor coordination, although not expressed in skeletal muscle, motoneurons, or spinal cord neurons. ANO2 is, however, present in Purkinje neurons of the cerebellum, and *Ano2* ^*−/−*^ mice display a mild form of ataxia combined with an impaired capability for motor learning (Zhang et al. 2015; Neureither et al. 2017). Moreover, a contribution to a motor phenotype may come from the striatum, as striatal low‐threshold spiking, neuropeptide Y‐positive interneurons express ANO2 and appear to require the channels for maintaining membrane potential oscillations of 3–7 Hz (Song et al. 2016). A striatal aspect of the *Ano2* ^*−/−*^ phenotype must await future investigation when conditional ANO2 knockouts become available. However, motor impairment appears not to be a problem for the *Ano2* ^*−/−*^ mice in our odor‐tracking tests, as the animals performed normally in pretesting (Laboras, SHIRPA, open field) and displayed normal explorative behavior throughout the 5 days of testing. ANO2 is also expressed in rod photoreceptor terminals (Stohr et al. 2009; Dauner et al. 2013), in dendrites of hippocampal pyramidal neurons (Huang et al. 2012), and in thalamocortical neurons (Ha et al. 2016). For the visual system, no *Ano2* ^*−/−*^ phenotype has been reported so far. ANO2 channels seem to be involved in the regulation of glutamate release from rod terminals under scotopic conditions (Dauner et al. 2013), but the contribution of ANO2 to retinal performance is not yet understood. In any case, our experiments were conducted under photopic conditions, and cone photoreceptor cells do not express ANO2 (Dauner et al. 2013). Moreover, in our control for visual tracking, where the animals could see the box containing the reward, both genotypes were able to rapidly find the reward, a finding that argues against impaired photopic vision in *Ano2* ^*−/−*^ mice. A hippocampal contribution to odor‐tracking performance still needs to be examined. However, in our and in other studies (Billig et al. 2011; Pietra et al. 2016), mice did not display any problems with odor learning, provided the odor concentrations were high enough. Indeed, *Ano2* ^*−/−*^ mice performed well in operant‐conditioning experiments based on odor learning. Problems for *Ano2* ^*−/−*^ mice did not originate from an inability to store odor information but from the fuzziness of the afferent signal at the threshold odor concentration of 0.03 mg/mL syringol. There is thus no evidence for a compromised odor‐memory system in *Ano2* ^*−/−*^ mice. Finally, thalamocortical neurons, which transmit sensory information from the ventrobasal thalamic nuclei to the cortex, express ANO2. These neurons display an ANO2‐mediated type of self‐inhibition that reduces their spike frequency during sensory stimulation (Ha et al. 2016). Interestingly, this study revealed that the knockdown of ANO2 in thalamocortical neurons increased the sensitivity to visceral, but not to acute pain. However, a link between these neurons and the olfactory phenotype of *Ano2* ^*−/−*^ mice is difficult to envisage, as olfaction is the one sensory modality that does not channel its afferent signals through primary thalamic nuclei toward the cortex. Instead, olfactory cortical areas (piriform, orbitofrontal, and entorhinal cortices) are directly supplied by the olfactory bulb, while the mediodorsal thalamic nuclei function as higher order processing units in the olfactory system (Tham et al. 2009; Courtiol and Wilson 2015). ANO2 expression has not been demonstrated for these nuclei. Taken together, the evidence presently available on ANO2 channels in the brain does not point to any central dysfunction involved in the tracking phenotype. Furthermore, for the interpretation of our results, one has to consider that the tracking phenotype emerges exclusively in naïve *Ano2* ^*−/−*^ mice. It is thus specifically associated with a cognitive state of the animal, not with general olfactory performance. Both genotypes have the same detection threshold and the same abilities for odor discrimination once they have learned to recognize an odor (Billig et al. 2011). It is, however, harder for *Ano2* ^*−/−*^ mice to learn when the odor is presented at threshold concentration. This highly specific phenotype of odor learning under challenging conditions is most likely unrelated to the motor system or the visual system. However, this point awaits verification by behavioral studies of conditional *Ano2* knockouts, with the gene ablation specifically targeted to ORNs.

The results of the present study are in accordance with published data on ANO2, and they resolve a controversy sparked by the observation that, in *Ano2* ^*−/−*^ mice, odor discrimination and odor detection thresholds were similar to wild‐type animals (Billig et al. 2011). This finding was obtained in operant‐conditioning experiments with mice trained to detect and distinguish specific odorants, and the results clearly revealed that the ANO2‐mediated amplification was not required for these tasks. In conditioning paradigms, mice first memorize target odors and then recognize these odors in detection or discrimination tests. The published experiments were thus performed with experienced *Ano2* ^*−/−*^ mice, and they led to the same conclusion as our study: Once an odor has been memorized as an olfactory object, it can be detected at threshold concentrations without amplification. The phenotype of *Ano2* ^*−/−*^ mice is specifically related to the learning and consolidation of odor objects for unfamiliar odors presented at threshold intensity. Consistent with this conclusion is the recently published observation that odor‐naïve *Ano2* ^*−/−*^ mice required more time than wild types to locate objects with unfamiliar odors until they learned to recognize them (Pietra et al. 2016). Interestingly, this study also revealed that ORNs in *Ano2* ^*−/−*^ mice had a reduced spontaneous firing activity in the absence of odor simulation, and that this reduced basal activity affected the wiring between olfactory epithelium and olfactory bulb. In wild‐type animals, axons of most ORNs that express the same odorant receptor coalesce onto four glomeruli, and this sparse patterning is promoted by the basal activity of ORNs (Lodovichi and Belluscio 2012; Lorenzon et al. 2015). In *Ano2* ^*−/−*^ mice, ORNs with the same receptor form additional glomeruli and thus alter the spatial representation of odors in the bulb (Pietra et al. 2016). Such changes in the neural odor map may create an additional impediment for odor information processing. This notion is, however, speculative, as experienced *Ano2* ^*−/−*^ mice detect and distinguish odorants just as well as wild types (Billig et al. 2011). Any possible significance of odor‐map changes for naïve mice still has to be examined. Additional information for understanding Cl^−^‐based signal amplification came from single‐ORN recordings. A recently developed method makes it possible to separate quantitatively cation currents from chloride currents during odor response (Li et al. 2016). The study demonstrated that the ratio of currents through Ca^2+^‐gated Cl^−^ channels and CNG channels, I_Cl_/I_cation_, increased at low odor concentrations, an observation consistent with our finding that ANO2‐mediated amplification is strongest at low stimulus intensities. Moreover, single‐ORN recordings from *Ano2* ^*−/−*^ mice produced prolonged responses to odor stimulation (Pietra et al. 2016). This effect may contribute to the reduced temporal resolution that we found in the EOG recording. Despite these points of consistency, single‐ORN recordings and EOG recordings should not be expected to match in every respect. ORNs obtained from dissociated olfactory epithelium may respond differently to odors when compared to ORNs contained in intact epithelial tissue with its separation of basolateral and mucosal solutions (Kaneko et al. 2004). Perireceptor factors, including mucosal ion concentrations and odor‐binding proteins, as well as paracrine effects of neuromodulation, may profoundly shape the afferent signal. While the precise relation between single‐ORN recordings and EOGs was, to our knowledge, never explored systematically, there are examples that the two methods may support inconsistent concepts. Particularly for complex transduction processes like adaptation, single‐ORN recordings offer only limited value for predicting the compound afferent signal and its central processing (Song et al. 2008; Cygnar et al. 2012; Stephan et al. 2012), a concern that may also apply to peripheral signal amplification. Isolated ORNs certainly offer better access to dissecting individual aspects of the transduction process, but EOG recordings are better suited for the study of afferent signals. For this reason, EOGs were employed in the present study to complement our behavioral studies.

In summary, our results suggest that the tracking performance of *Ano2* ^*−/−*^ mice is impaired because their olfactory epithelium operates with reduced sensitivity and temporal resolution. While these deficits do not compromise the detection of familiar or intense smells, they incapacitate sensory function when odor stimuli are both weak and novel. The tracking deficits of syringol‐naïve *Ano2* ^*−/−*^ mice at threshold stimulus intensity indicate that the animals fail to construct an olfactory object to represent the syringol scent. Apparently, an amplified output of ORNs is required for this learning process. Without amplification, the olfactory epithelium of *Ano2* ^*−/−*^ mice generates inaccurate odor information, too blurred to serve as useful template for the generation of odor objects. Taking this concept to the level of sensory evolution leads to the hypothesis that the selective pressure that stabilizes the expression of ANO2 in mammalian ORNs is the animals’ need to construct odor objects from weak scents. As the interplay between CNG channels and Ca^2+^‐gated Cl^−^ channels was found also in ORNs from freshwater fish (Sato and Suzuki 2000), amphibians (Kleene and Gesteland 1991), and reptiles (Kashiwayanagi et al. 1996), as well as in the pheromone‐sensitive neurons of the vomeronasal organ (Kim et al. 2011; Dibattista et al. 2012; Amjad et al. 2015), it may represent a general mechanism for tracking faint chemical stimuli in vertebrates.

## Conflict of Interest

The authors declare that they have no conflict of interest.

## Data Accessibility

## Supporting information




**Movie S1:** Wild type: Successful tracking performance.

**Movie S2:** ANO2 knockout: Unsuccessful tracking performance.

## References

[phy213373-bib-0001] Amjad, A. , A. Hernandez‐Clavijo , S. Pifferi , D. K. Maurya , A. Boccaccio , J. Franzot , et al. 2015 Conditional knockout of TMEM16A/anoctamin1 abolishes the calcium‐activated chloride current in mouse vomeronasal sensory neurons. J. Gen. Physiol. 145:285–301. 25779870 10.1085/jgp.201411348 PMC4380210

[phy213373-bib-0002] Barnes, D. C. , R. D. Hofacer , A. R. Zaman , R. L. Rennaker , and D. A. Wilson . 2008 Olfactory perceptual stability and discrimination. Nat. Neurosci. 11:1378–1380. 18978781 10.1038/nn.2217 PMC2682180

[phy213373-bib-0003] Bhandawat, V. , J. Reisert , and K. W. Yau . 2005 Elementary response of olfactory receptor neurons to odorants. Science 308:1931–1934. 15976304 10.1126/science.1109886 PMC2957801

[phy213373-bib-0004] Bhandawat, V. , J. Reisert , and K. W. Yau . 2010 Signaling by olfactory receptor neurons near threshold. Proc. Natl Acad. Sci. USA 107:18682–18687. 20930117 10.1073/pnas.1004571107 PMC2972989

[phy213373-bib-0005] Billig, G. M. , B. Pal , P. Fidzinski , and T. J. Jentsch . 2011 Ca^2+^‐activated Cl^−^ currents are dispensable for olfaction. Nat. Neurosci. 14:763–769. 21516098 10.1038/nn.2821

[phy213373-bib-0006] Buck, L. , and R. Axel . 1991 A novel multigene family may encode odorant receptors: a molecular basis for odor recognition. Cell 65:175–187. 1840504 10.1016/0092-8674(91)90418-x

[phy213373-bib-0007] Burdock, G. A. 2010 Pp. 451–452. Fenaroli′s handbook of flavor ingredients. CRC Press, New York, NY.

[phy213373-bib-0008] Chapuis, J. , and D. A. Wilson . 2011 Bidirectional plasticity of cortical pattern recognition and behavioral sensory acuity. Nat. Neurosci. 15:155–161. 22101640 10.1038/nn.2966 PMC3245808

[phy213373-bib-0009] Coppola, D. M. , C. T. Waggener , S. M. Radwani , and D. A. Brooks . 2013 An electroolfactogram study of odor response patterns from the mouse olfactory epithelium with reference to receptor zones and odor sorptiveness. J. Neurophysiol. 109:2179–2191. 23343905 10.1152/jn.00769.2012

[phy213373-bib-0010] Courtiol, E. , and D. A. Wilson . 2015 The olfactory thalamus: unanswered questions about the role of the mediodorsal thalamic nucleus in olfaction. Front. Neural Circuits 9:49. 26441548 10.3389/fncir.2015.00049 PMC4585119

[phy213373-bib-0011] Courtiol, E. , and D. A. Wilson . 2017 The olfactory mosaic: bringing an olfactory network together for odor perception. Perception 46:320–332. 27687814 10.1177/0301006616663216 PMC5362339

[phy213373-bib-0012] Cygnar, K. D. , S. E. Collins , C. H. Ferguson , C. Bodkin‐Clarke , and H. Zhao . 2012 Phosphorylation of adenylyl cyclase III at serine1076 does not attenuate olfactory response in mice. J. Neurosci. 32:14557–14562. 23077041 10.1523/JNEUROSCI.0559-12.2012 PMC3490616

[phy213373-bib-0013] Dauner, K. , C. Mobus , S. Frings , and F. Mohrlen . 2013 Targeted expression of anoctamin calcium‐activated chloride channels in rod photoreceptor terminals of the rodent retina. Invest. Ophthalmol. Vis. Sci. 54:3126–3136. 23557741 10.1167/iovs.13-11711

[phy213373-bib-0014] Dibattista, M. , A. Amjad , D. K. Maurya , C. Sagheddu , G. Montani , R. Tirindelli , et al. 2012 Calcium‐activated chloride channels in the apical region of mouse vomeronasal sensory neurons. J. Gen. Physiol. 140:3–15. 22732308 10.1085/jgp.201210780 PMC3382724

[phy213373-bib-0015] Dibattista, M. , S. Pifferi , A. Boccaccio , A. Menini , and J. Reisert . 2017 The long tale of the calcium activated Cl^−^ Channels in olfactory transduction. Channels (Austin). https://doi.org/10.1080/19336950.2017.1307489. [Epub ahead of print]. 10.1080/19336950.2017.1307489 PMC5626357 28301269

[phy213373-bib-0016] Dzeja, C. , V. Hagen , U. B. Kaupp , and S. Frings . 1999 Ca^2+^ permeation in cyclic nucleotide‐gated channels. EMBO J. 18:131–144. 9878057 10.1093/emboj/18.1.131 PMC1171109

[phy213373-bib-0017] Fukunaga, I. , M. Berning , M. Kollo , A. Schmaltz , and A. T. Schaefer . 2012 Two distinct channels of olfactory bulb output. Neuron 75:320–329. 22841316 10.1016/j.neuron.2012.05.017

[phy213373-bib-0018] Gadziola, M. A. , K. A. Tylicki , D. L. Christian , and D. W. Wesson . 2015 The olfactory tubercle encodes odor valence in behaving mice. J. Neurosci. 35:4515–4527. 25788670 10.1523/JNEUROSCI.4750-14.2015 PMC6605138

[phy213373-bib-0019] Ghatpande, A. S. , and J. Reisert . 2011 Olfactory receptor neuron responses coding for rapid odour sampling. J. Physiol. (Lond) 589:2261–2273. 21486768 10.1113/jphysiol.2010.203687 PMC3098702

[phy213373-bib-0020] Gottfried, J. A. 2010 Central mechanisms of odour object perception. Nat. Rev. Neurosci. 11:628–641. 20700142 10.1038/nrn2883 PMC3722866

[phy213373-bib-0021] Ha, G. E. , J. Lee , H. Kwak , K. Song , J. Kwon , S. Y. Jung , et al. 2016 The Ca^2+^‐activated chloride channel anoctamin‐2 mediates spike‐frequency adaptation and regulates sensory transmission in thalamocortical neurons. Nat. Comm. 7:13791. 10.1038/ncomms13791 PMC5187435 27991499

[phy213373-bib-0022] Huang, W. C. , S. Xiao , F. Huang , B. D. Harfe , Y. N. Jan , and L. Y. Jan . 2012 Calcium‐activated chloride channels (CaCCs) regulate action potential and synaptic response in hippocampal neurons. Neuron 74:179–192. 22500639 10.1016/j.neuron.2012.01.033 PMC3329964

[phy213373-bib-0023] Kaneko, H. , I. Putzier , S. Frings , U. B. Kaupp , and T. Gensch . 2004 Chloride accumulation in mammalian olfactory sensory neurons. J. Neurosci. 24:7931–7938. 15356206 10.1523/JNEUROSCI.2115-04.2004 PMC6729923

[phy213373-bib-0024] Kashiwayanagi, M. , H. Kawahara , K. Kanaki , F. Nagasawa , and K. Kurihara . 1996 Ca^2+^ and Cl–dependence of the turtle olfactory response to odorants and forskolin. Comp. Biochem. Physiol. A 115:43–52. 10.1016/0300-9629(95)02139-6 8858838

[phy213373-bib-0025] Kim, S. , L. Ma , and C. R. Yu . 2011 Requirement of calcium‐activated chloride channels in the activation of mouse vomeronasal neurons. Nat. Comm. 2:365. 10.1038/ncomms1368 PMC3156823 21694713

[phy213373-bib-0026] Kleene, S. J. 1993 Origin of the chloride current in olfactory transduction. Neuron 11:123–132. 8393322 10.1016/0896-6273(93)90276-w

[phy213373-bib-0027] Kleene, S. J. 1995 Block by external calcium and magnesium of the cyclic‐nucleotide‐activated current in olfactory cilia. Neuroscience 66:1001–1008. 7651604 10.1016/0306-4522(94)00634-h

[phy213373-bib-0028] Kleene, S. J. , and R. C. Gesteland . 1991 Calcium‐activated chloride conductance in frog olfactory cilia. J. Neurosci. 11:3624–3629. 1941099 10.1523/JNEUROSCI.11-11-03624.1991 PMC6575529

[phy213373-bib-0029] Kumar, K. R. , and G. Archunan . 1999 Influence of the stage of the cycle on olfactory sensitivity in laboratory mice. Indian J. Exp. Biol. 37:317–318. 10641165

[phy213373-bib-0030] Kurahashi, T. , and K. W. Yau . 1993 Co‐existence of cationic and chloride components in odorant‐induced current of vertebrate olfactory receptor cells. Nature 363:71–74. 7683113 10.1038/363071a0

[phy213373-bib-0031] Li, R. C. , Y. Ben‐Chaim , K. W. Yau , and C. C. Lin . 2016 Cyclic‐nucleotide‐gated cation current and Ca^2+^‐activated Cl current elicited by odorant in vertebrate olfactory receptor neurons. Proc. Natl Acad. Sci. USA 113:11078–11087. 27647918 10.1073/pnas.1613891113 PMC5056050

[phy213373-bib-0032] Lodovichi, C. , and L. Belluscio . 2012 Odorant receptors in the formation of the olfactory bulb circuitry. Physiology (Bethesda) 27:200–212. 22875451 10.1152/physiol.00015.2012

[phy213373-bib-0033] Lorenzon, P. , N. Redolfi , M. J. Podolsky , I. Zamparo , S. A. Franchi , G. Pietra , et al. 2015 Circuit formation and function in the olfactory bulb of mice with reduced spontaneous afferent activity. J. Neurosci. 35:146–160. 25568110 10.1523/JNEUROSCI.0613-14.2015 PMC6605243

[phy213373-bib-0034] Lowe, G. , and G. H. Gold . 1993 Nonlinear amplification by calcium‐dependent chloride channels in olfactory receptor cells. Nature 366:283–286. 8232590 10.1038/366283a0

[phy213373-bib-0035] Nakamura, T. , and G. H. Gold . 1987 A cyclic nucleotide‐gated conductance in olfactory receptor cilia. Nature 325:442–444. 10.1038/325442a0 3027574

[phy213373-bib-0036] Neureither, F. , K. Ziegler , C. Pitzer , S. Frings , and F. Mohrlen . 2017 Impaired motor coordination and learning in mice lacking anoctamin 2 calcium‐gated chloride channels. Cerebellum. https://doi.org/10.1007/s12311-017-0867-4. [Epub ahead of print]. 10.1007/s12311-017-0867-4 PMC5717130 28536821

[phy213373-bib-0037] Nickell, W. T. , N. K. Kleene , R. C. Gesteland , and S. J. Kleene . 2006 Neuronal chloride accumulation in olfactory epithelium of mice lacking NKCC1. J. Neurophysiol. 95:2003–2006. 16319203 10.1152/jn.00962.2005 PMC1379662

[phy213373-bib-0038] Nickell, W. T. , N. K. Kleene , and S. J. Kleene . 2007 Mechanisms of neuronal chloride accumulation in intact mouse olfactory epithelium. J. Physiol. (Lond.) 583:1005–1020. 17656441 10.1113/jphysiol.2007.129601 PMC2277205

[phy213373-bib-0039] Pietra, G. , M. Dibattista , A. Menini , J. Reisert , and A. Boccaccio . 2016 The Ca^2+^‐activated Cl^−^ channel TMEM16B regulates action potential firing and axonal targeting in olfactory sensory neurons. J. Gen. Physiol. 148:293–311. 27619419 10.1085/jgp.201611622 PMC5037344

[phy213373-bib-0040] Reisert, J. , and H. R. Matthews . 1998 Na^+^‐dependent Ca^2+^ extrusion governs response recovery in frog olfactory receptor cells. J. Gen. Physiol. 112:529–535. 9806962 10.1085/jgp.112.5.529 PMC2229439

[phy213373-bib-0041] Reisert, J. , P. J. Bauer , K. W. Yau , and S. Frings . 2003 The Ca‐activated Cl channel and its control in rat olfactory receptor neurons. J. Gen. Physiol. 122:349–363. 12939394 10.1085/jgp.200308888 PMC2234486

[phy213373-bib-0042] Reisert, J. , J. Lai , K. W. Yau , and J. Bradley . 2005 Mechanism of the excitatory Cl^−^ response in mouse olfactory receptor neurons. Neuron 45:553–561. 15721241 10.1016/j.neuron.2005.01.012 PMC2877386

[phy213373-bib-0043] Ressler, K. J. , S. L. Sullivan , and L. B. Buck . 1993 A zonal organization of odorant receptor gene expression in the olfactory epithelium. Cell 73:597–609. 7683976 10.1016/0092-8674(93)90145-g

[phy213373-bib-0044] Rogers, D. C. , E. M. Fisher , S. D. Brown , J. Peters , A. J. Hunter , and J. E. Martin . 1997 Behavioral and functional analysis of mouse phenotype: SHIRPA, a proposed protocol for comprehensive phenotype assessment. Mamm. Genome 8:711–713. 9321461 10.1007/s003359900551

[phy213373-bib-0045] Sagheddu, C. , A. Bocaccio , M. Dibattista , G. Montani , R. Tirindelli , and A. Menini . 2010 Calcium concentration jumps reveal dynamic ion selectivity of calcium‐activated chloride currents in mouse olfactory sensory neurons and TMEM16b‐transfected HEK 293T cells. J. Physiol. (Lond.) 588:4189–4204. 20837642 10.1113/jphysiol.2010.194407 PMC3002450

[phy213373-bib-0046] Sato, K. , and N. Suzuki . 2000 The contribution of a Ca^2+^‐activated Cl^−^ conductance to amino‐acid‐induced inward current responses of ciliated olfactory neurons of the rainbow trout. J. Exp. Biol. 203:253–262. 10607535 10.1242/jeb.203.2.253

[phy213373-bib-0047] Schroeder, B. C. , T. Cheng , Y. N. Jan , and Jan L. Y. . 2008 Expression cloning of TMEM16A as a calcium‐activated chloride channel subunit. Cell 134:1019–1029. 18805094 10.1016/j.cell.2008.09.003 PMC2651354

[phy213373-bib-0048] Scott, J. W. , and P. E. Scott‐Johnson . 2002 The electroolfactogram: a review of its history and uses. Microsc. Res. Tech. 58:152–160. 12203693 10.1002/jemt.10133

[phy213373-bib-0049] Shusterman, R. , M. C. Smear , A. A. Koulakov , and D. Rinberg . 2011 Precise olfactory responses tile the sniff cycle. Nat. Neurosci. 14:1039–1044. 21765422 10.1038/nn.2877 PMC13348895

[phy213373-bib-0050] Song, Y. , K. D. Cygnar , B. Sagdullaev , M. Valley , S. Hirsh , A. Stephan , et al. 2008 Olfactory CNG channel desensitization by Ca^2+^/CaM via the B1b subunit affects response termination but not sensitivity to recurring stimulation. Neuron 58:374–386. 18466748 10.1016/j.neuron.2008.02.029 PMC2587172

[phy213373-bib-0051] Song, S. C. , J. A. Beatty , and C. J. Wilson . 2016 The ionic mechanism of membrane potential oscillations and membrane resonance in striatal LTS interneurons. J. Neurophysiol. 116:1752–1764. 27440246 10.1152/jn.00511.2016 PMC5144687

[phy213373-bib-0052] Stephan, A. B. , E. Y. Shum , S. Hirsh , K. D. Cygnar , J. Reisert , and H. Zhao . 2009 ANO2 is the cilial calcium‐activated chloride channel that may mediate olfactory amplification. Proc. Natl Acad. Sci. USA 106:11776–11781. 19561302 10.1073/pnas.0903304106 PMC2702256

[phy213373-bib-0053] Stephan, A. B. , S. Tobochnik , M. Dibattista , C. M. Wall , J. Reisert , and H. Zhao . 2012 The Na^+^/Ca^2+^ exchanger NCKX4 governs termination and adaptation of the mammalian olfactory response. Nat. Neurosci. 15:131–137. 10.1038/nn.2943 PMC3245797 22057188

[phy213373-bib-0054] Stohr, H. , J. B. Heisig , P. M. Benz , S. Schoberl , V. M. Milenkovic , O. Strauss , et al. 2009 TMEM16B, A novel protein with calcium‐dependent chloride channel activity, associates with a presynaptic protein complex in photoreceptor terminals. J. Neurosci. 29:6809–6818. 19474308 10.1523/JNEUROSCI.5546-08.2009 PMC6665584

[phy213373-bib-0055] Tham, W. W. , R. J. Stevenson , and L. A. Miller . 2009 The functional role of the medio dorsal thalamic nucleus in olfaction. Brain Res. Rev. 62:109–126. 19800366 10.1016/j.brainresrev.2009.09.007

[phy213373-bib-0056] Tsanov, M. , E. Chah , R. Reilly , and S. M. O'Mara . 2014 Respiratory cycle entrainment of septal neurons mediates the fast coupling of sniffing rate and hippocampal theta rhythm. Eur. J. Neurosci. 39:957–974. 24329896 10.1111/ejn.12449 PMC4165309

[phy213373-bib-0057] Wilson, D. A. , and R. J. Stevenson . 2003 The fundamental role of memory in olfactory perception. Trends Neurosci. 26:243–247. 12744840 10.1016/S0166-2236(03)00076-6

[phy213373-bib-0058] Wilson, D. A. , and R. M. Sullivan . 2011 Cortical processing of odor objects. Neuron 72:506–519. 22099455 10.1016/j.neuron.2011.10.027 PMC3223720

[phy213373-bib-0059] Yeshurun, Y. , and N. Sobel . 2010 An odor is not worth a thousand words: from multidimensional odors to unidimensional odor objects. Annu. Rev. Psychol. 61:219–241. 19958179 10.1146/annurev.psych.60.110707.163639

[phy213373-bib-0060] Zhang, W. , S. Schmelzeisen , D. Parthier , S. Frings , and F. Mohrlen . 2015 Anoctamin calcium‐activated chloride channels may modulate inhibitory transmission in the cerebellar cortex. PLoS ONE 10:e0142160. 26558388 10.1371/journal.pone.0142160 PMC4641602

